# A Rare Pediatric Presentation: Concurrent Detection of All Five Hepatitis B Virus (HBV) Serological Markers

**DOI:** 10.3390/jcm15082823

**Published:** 2026-04-08

**Authors:** Menglan Zhang, Wensheng Li, Zhengxiang Gao, Chenxi Liu

**Affiliations:** 1 Department of Laboratory Medicine, West China Second University Hospital, Sichuan University, Chengdu 610041, China; adazml@163.com (M.Z.); 18310959352@163.com (W.L.); gaozx84@163.com (Z.G.); 2 Key Laboratory of Birth Defects and Related Diseases of Women and Children, Ministry of Education, Sichuan University, Chengdu 610041, China

**Keywords:** case report, hepatitis B virus, serological markers

## Abstract

**Background:** This case report presents a 12-year-old male with vertically transmitted chronic hepatitis B virus (HBV) infection, exhibiting a rare pan-reactive serological profile (concurrent HBsAg, HBsAb, HBeAg, HBeAb, and HBcAb positivity) alongside fluctuating low-level viremia (HBV DNA: 1.06 × 10^2^ IU/mL to undetectable). Rigorous exclusion of technical artifacts confirmed the authenticity of this atypical serologic pattern, observed in <0.001% of the general population. **Methods:** Liver biopsy and immunohistochemical staining were performed to evaluate hepatic inflammation and fibrosis. HBV serological markers and viral load were quantified using commercial diagnostic kits, with longitudinal monitoring for 18 months. **Results:** Liver biopsy revealed Grade 2 inflammation with focal HBsAg/HBcAg expression, supporting immune-active chronic hepatitis B (CHB) despite partial seroconversion. The patient’s clinical course highlights key challenges in pediatric HBV management: (1) delayed immune reconstitution (18-month longitudinal HBeAg/HBeAb dynamics), (2) non-linear virologic-ALT correlation, and (3) diagnostic ambiguity in pan-positive serology—potentially reflecting S-gene escape mutants or transitional immune responses. Initiation of tenofovir disoproxil fumarate (TDF) achieved sustained virologic suppression, underscoring the importance of early antiviral therapy in pediatric CHB with atypical markers. **Conclusions:** This case provides preliminary insights into the complex interplay between viral evolution and immature host immunity, advocating for refined monitoring protocols integrating high-sensitivity HBV DNA, quantitative serology, and non-invasive fibrosis assessment in pediatric HBV care.

## 1. Introduction

Hepatitis B virus (HBV) infection remains a critical global public health challenge, with persistent threats to human health. Surveillance findings through 2019 indicate that chronic HBV-related liver diseases have affected an estimated 46.5 million children and adolescents aged 0–19 years globally [1]. Of particular concern is the infection burden among young children. Model-based estimates from the Polaris Observatory Collaborators reveal that in 2022, the global prevalence of hepatitis B surface antigen (HBsAg) in children under 5 years remained at 0.7%, corresponding to approximately 5.6 million infected pediatric cases [2]. These epidemiological patterns underscore the imperative for sustained intensification of prevention strategies, particularly in enhancing perinatal transmission-blocking protocols and maintaining high coverage rates of childhood immunization programs.

Since the nationwide implementation of neonatal hepatitis B vaccination in China’s Expanded Program on Immunization in 1992, the HBV infection rate among children has undergone a substantial decline. Nevertheless, complete prevention of mother-to-child transmission (MTCT) remains challenging, despite the standardized perinatal prophylaxis protocol being widely adopted. Regional disparities in healthcare resources and individual variations in host immune responses contribute to residual MTCT risks. A 2024 multi-center observational cohort study reported the overall HBV MTCT rate of 0.23% [3]. Notably, China’s large population base sustains a considerable HBV carrier pool. From 2013 to 2020, the overall prevalence of HBsAg among pregnant women in mainland China was 6.27% (95% CI: 6.26–6.28%) [4]. This persistent challenge necessitates refined prevention strategies, particularly in optimizing precision interventions for pediatric hepatitis B control under the current three-child policy framework.

In contrast to adults, pediatric HBV infection carries a significantly higher risk of chronic progression. Perinatal and infantile infections demonstrate chronicity rates approaching 90%, compared to 20–30% in children aged 1–5 years [5]. While the natural history of chronic HBV infection has been extensively characterized in adults, robust longitudinal data remain scarce for pediatric populations, particularly those under 12 years of age.

Distinct from the well-defined four-phase natural history classification in adults, pediatric infections predominantly cluster within the hepatitis B e antigen (HBeAg)-positive chronic HBV infection phase and HBeAg-positive chronic hepatitis B (CHB) phase. However, the proportional distribution across natural history phases remains incompletely characterized. A 2023 Chinese retrospective cohort study investigating pediatric chronic HBV carriers demonstrated distinct phase distribution patterns, with HBeAg-positive chronic HBV infection constituting the predominant phenotype at 40.5%, followed by HBeAg-positive CHB at 16.3%. The cohort further revealed HBeAg-negative chronic HBV infection in 10.5% of cases and HBeAg-negative CHB in 3.5%, while a substantial proportion (29.2%) remained unclassifiable within conventional natural history categories, underscoring the unique challenges in staging pediatric HBV infections compared to adult populations [6].

Evidence indicates an annual spontaneous HBeAg seroclearance rate < 5% and HBsAg seroclearance rate < 1% in pediatric chronic HBV carriers [7]. Meta-analyses identify infection age and baseline alanine aminotransferase (ALT) levels as key determinants of HBeAg clearance. Although chronic HBV infection in childhood is a benign process, 3% to 5% of children progress to cirrhosis before adulthood, and 0.01% to 0.03% of children progress to liver cancer. Considering the whole life cycle, the incidence rate of liver cancer rises to 9% to 24% every year, and the incidence rate of liver cirrhosis rises to 2% to 3% [8].

The case elucidates the mechanisms underlying concurrent seropositivity of all HBV markers and viral load fluctuations through longitudinal monitoring of a rare clinical case of vertically transmitted chronic hepatitis B in a pediatric patient.

## 2. Case Presentation

**Figure 1:**
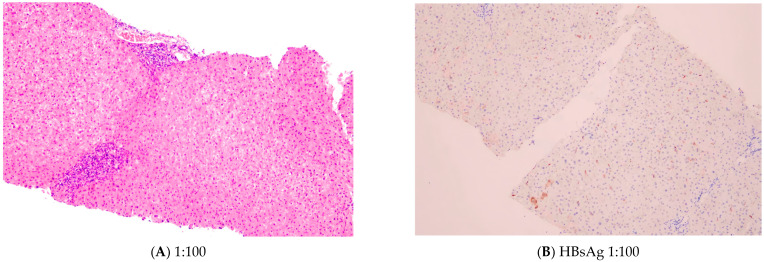
Liver biopsy findings in the presented case. (**A**) Hematoxylin and eosin (H&E) staining (100×) demonstrates preserved hepatic architecture with focal hepatocyte regeneration, occasional spotty necrosis, and mild interface activity. Portal tracts exhibit sparse lymphomonocytic infiltration. (**B**) Immunohistochemistry showed scattered HBsAg-positive hepatocytes (1%) and HBeAg expression (8%).

A 12-year-old male with chronic active HBV infection presented for outpatient follow-up, where an atypical pan-positive serological profile (concurrent reactivity of HBsAg, hepatitis B surface antibody [HBsAb], HBeAg, hepatitis B e antibody [HBeAb], and hepatitis B core total antibody [HBcAb(T)]) was identified (Table 1). Technical artifacts were excluded through rigorous validation: specimen integrity assessment (no hemolysis, lipemia, or clots; re-centrifugation confirmed absence of microclots), instrument calibration verification (stable quality control charts, normal environmental parameters), and repeat testing consistency. Clinical history review revealed initial HBV diagnosis 18 months prior (external lab findings: HBsAg+/HBsAb+/HBeAg+/HBeAb−/HBcAb(T)+, HBV DNA 5.59 × 10^5^ IU/mL), with mild transaminase elevation ALT 45 U/L, aspartate aminotransferase [AST] 43 U/L, prealbumin 194 mg/L) and absence of chronic liver disease stigmata. Neonatal HBV vaccination (without hepatitis B immunoglobulin [HBIG]) and formula feeding were documented. Maternal HBV history included HBeAg-positive chronic HBV (diagnosed 2004, briefly treated with herbal therapy without sustained virologic response), transitioning to HBeAg-negative chronic HBV (HBsAg+/HBeAb+/HBcAb(T)+) by August 2023. Physical examination demonstrated stable vital signs, mild scleral icterus, and a tender liver edge 2 cm below the costal margin, without chronic liver disease signs (spider angiomas, splenomegaly). Diagnostic workup excluded hepatitis A/C/D/E co-infections and revealed an unremarkable abdominal ultrasound. Ultrasound-guided percutaneous liver biopsy showed preserved lobular architecture with focal hepatocyte necrosis (Grade 1–2 inflammation), mild portal lymphocytic infiltration, and Stage 1 fibrosis (Ishak staging system). Immunohistochemistry detected focal HBsAg (1% positivity) and HBcAg (8% positivity), and in situ hybridization for Epstein–Barr virus-encoded small RNA (EBER) was negative, ruling out Epstein–Barr virus (EBV)-driven pathogenesis (Figure 1). The patient was diagnosed with chronic active hepatitis B (Grade 2 inflammation, Stage 1 fibrosis) and initiated on tenofovir disoproxil fumarate (TDF) with hepatoprotective agents. Post-discharge follow-up demonstrated sustained virologic suppression (undetectable HBV DNA) and biochemical stability under TDF therapy, with regular monitoring of hepatic/renal function and electrolytes since August 2023 (Table 2).

## 3. Materials and Methods

Clinical data including medical history, physical examination, laboratory tests, imaging findings, treatment process, and follow-up information were collected from the patient’s medical records at West China Second University Hospital of Sichuan University.

Routine blood tests and biochemical analysis were performed according to the standard clinical laboratory protocols of our hospital. For HBV-related serological markers, commercial diagnostic kits from Roche Diagnostics (Basel, Switzerland) were used. The specific normal ranges, units, and corresponding titers for each HBV marker are presented in Table 1. In particular, HBsAg detection utilized monoclonal and polyclonal antibodies targeting the ‘a’ determinant (major immunodominant epitope of HBsAg) and other subtype-specific determinants. Anti-HBs detection employed a mixture of native and recombinant HBsAg antigens (subtypes ad/ay) to recognize antibodies against the common a determinant and subtype-specific epitopes. HBV DNA quantification was performed using a commercial fluorescent PCR kit from Daan Gene Co., Ltd (Guangzhou, China), which targets a conserved region of the HBV genome and is validated for the detection of genotypes B, C, D, B/C mixtures, and C/D mixtures (common Asian genotypes).

Ultrasound-guided percutaneous liver biopsy was performed, and subsequent immunohistochemical staining was conducted following standard pathological procedures.

## 4. Case Discussion

This case represents a classic instance of MTCT, the predominant route of pediatric HBV infection in China, accounting for over 90% of childhood cases [9]. Vertical transmission risks persist at 5–10% even with combined immunoprophylaxis (hepatitis B vaccine and HBIG), particularly when maternal HBeAg-positivity coincides with high viral load [10,11]. Longitudinal serological profiling over an 18-month follow-up revealed a rare pan-reactive pattern. Notable trends included significant declines in HBsAg, HBsAb, HBeAg, and HBeAb titers, accompanied by a progressive rise in HBeAb levels (Table 1).

The emergence of HBeAb typically correlates with HBeAg seroconversion, reflecting partial activation of the host immune response against HBV. In natural disease progression, immune-mediated viral clearance is marked by HBeAg disappearance, HBeAb seropositivity, and a concomitant decline in viral load, often accompanied by transient ALT elevation. However, pediatric patients exhibit distinct immunological constraints: spontaneous HBeAg seroclearance occurs at an annual rate of 2–5%—significantly lower than in adults—with younger age correlating with more pronounced immune tolerance [7]. During adolescence, when immune activation probabilities increase, some experience HBeAg reversion post-seroconversion, underscoring the fragile immune equilibrium in pediatric chronic HBV infection [12].

This pan-reactive pattern, occurring in <0.001% of cases (3/21,747 in one cohort), suggests a complex virologic state after excluding laboratory artifacts [13]. The concurrent seropositivity of all five hepatitis B markers may be associated with multiple potential mechanisms involving virological, immunological, and clinical peculiarities. One plausible hypothesis is the emergence of viral S-gene mutations, particularly in the HBsAg ‘a’ determinant—the major immunodominant epitope of HBsAg. Mutations in this region may alter HBsAg conformation, rendering pre-existing neutralizing antibodies (HBsAb) ineffective against mutant strains, thereby sustaining antigen–antibody coexistence [14]. This is consistent with the technical characteristics of the detection kits used, which are validated for identifying common ‘a’ determinant mutants. Alternatively, during serological transition phases—such as the shift from HBeAg-positive to HBeAg-negative status or acute infection resolution—temporal discordance between HBeAg clearance and HBeAb production can transiently manifest pan-positive serology, as a potential explanation for the observed phenomenon in this patient. HBeAg seroconversion is a critical milestone reflecting clinical improvement, whether spontaneous or treatment-induced. Immune dysregulation (e.g., autoimmune disorders, human immunodeficiency virus coinfection) may disrupt antigen–antibody homeostasis, while hepatitis C virus or EBV coinfection might induce false-positive results through epitope cross-reactivity in serological assays. Furthermore, atypical immune responses in occult HBV infection (characterized by undetectable serum HBV DNA despite ongoing intrahepatic replication) or vaccine breakthrough infections (coexistence of vaccine-induced HBsAb and wild-type/variant HBsAg) can produce paradoxical serological profiles, highlighting the necessity for comprehensive molecular and clinical correlation in diagnostic interpretation. Dynamic equilibrium between viral replication and host immunity warrants longitudinal monitoring of HBV DNA, liver function, and imaging. Importantly, rigorous validation (repeat testing, specimen integrity assessment, instrument calibration) excluded technical artifacts as the cause, supporting the clinical authenticity of this pattern. It should be noted that these mechanistic interpretations remain speculative without direct molecular evidence from this patient, due to the unavailability of original patient samples.

Longitudinal monitoring of HBV DNA and ALT levels demonstrated dynamic virological patterns characterized by an initial decline in viral load from baseline levels of 1.06 × 10^2^ IU/mL (indicative of low-level replication) to undetectable thresholds (<100 IU/mL), with subsequent intermittent detection of low-positive viral loads. The observed non-linear correlation between ALT elevation and HBV DNA dynamics may reflect complex interactions between viral replication kinetics and the developing host immune response- consistent with prior observations in pediatric CHB [6]. In immunotolerant pediatric patients exhibiting hallmark features of high HBV DNA titers (>10^6^–10^8^ IU/mL), persistent HBeAg positivity, and consistently normal ALT values, the substantial viral load variability (e.g., fluctuations spanning 10^5^–10^8^ IU/mL) may stem from multifactorial mechanisms. These include incomplete immune tolerance states marked by sporadic weak activation of natural killer (NK) cells or non-antigen-specific T-cell responses, epigenetic modifications regulating covalently closed circular DNA (cccDNA) transcriptional activity, and non-immune contributors such as hepatocyte metabolic reprogramming during growth phases, pubertal endocrine alterations, or transient innate immune activation triggered by intercurrent infections. Crucially, these immunotolerant-phase fluctuations remain distinct from immune-active hepatitis by maintaining ALT levels within normal limits (≤2× upper limit of normal) and lacking histological evidence of necroinflammatory activity.

The decision to initiate TDF therapy in this patient was guided by multiple lines of clinical evidence: (1) histopathological findings of Grade 2 inflammation and Stage 1 fibrosis (Ishak staging), indicating immune-active CHB; (2) persistent low-level viremia, which is associated with progressive liver injury in pediatric populations over long-term follow-up; (3) the patient’s age (12 years), which aligns with pediatric HBV treatment guidelines recommending antiviral therapy for immune-active CHB regardless of age [15]; and (4) the absence of contraindications to TDF (normal renal function, no history of bone disease). Post-treatment follow-up demonstrated sustained virologic suppression and biochemical stability, confirming the efficacy of TDF in this patient with atypical serology.

For similar pediatric patients with pan-reactive HBV serology, the standardized monitoring protocol involves implementing high-sensitivity HBV DNA quantification at 3–6 month intervals, complemented by serial measurements of quantitative HBeAg/HBsAg titers, longitudinal ALT tracking, and non-invasive fibrosis staging through modalities such as transient elastography [16]. Liver biopsy should be considered for patients with unclear disease activity (e.g., normal ALT but persistent viremia) to guide treatment decisions, and longitudinal follow-up into adolescence is essential, as immune activation during this period may alter serological patterns or disease trajectory. With the advancement of high-sensitivity molecular assays and enhanced reagent specificity, contemporary diagnostic platforms are increasingly identifying atypical serological profiles previously undetectable by conventional methods. These developments necessitate a multidimensional analytical framework in clinical practice, wherein precise therapeutic decision-making requires comprehensive integration of dynamic virological parameters (viral load kinetics), evolving serological markers (antigen/antibody seroconversion patterns), and histopathological correlates (inflammation/fibrosis grading).

This case underscores the complexity of HBV seroconversion dynamics in pediatric patients and highlights the necessity of multidisciplinary evaluation to decipher rare serologic patterns. Continuous technological advancements will likely unveil novel virologic profiles, emphasizing the importance of rigorous clinical interpretation. While the single-patient nature of this report limits the generalizability of our findings, and the absence of HBV genomic sequencing data prevents direct confirmation of hypotheses regarding S-gene mutations or immune-escape variants, the rare clinical phenotype and detailed longitudinal data still provide valuable practical insights into the diagnosis and management of atypical pediatric CHB—an entity that remains poorly characterized in the current literature. These findings provide preliminary evidence for deciphering atypical serological patterns in China, where non-classical HBV profiles increasingly challenge conventional diagnostic paradigms.

In conclusion, the clinical course of this patient underscores the importance of longitudinal monitoring and timely antiviral therapy for immune-active pediatric CHB, even with low-level viremia. Future studies with larger cohorts and comprehensive molecular analyses are needed to further explore the underlying mechanisms of such atypical serological profiles.

## Figures and Tables

**Table 1:** Serological profile of hepatitis B virus markers in the case patient.

Test Date(yyyy-mm)	2023-08	2024-07	2024-10	2025-04
HBsAg, COI (<1.0)	140.00 +	114.00 +	54.30 +	26.00 +
HBsAb, IU/L (<10)	220.00 +	74.30 +	79.80 +	27.40 +
HBeAg, COI (<1.0)	1763.00 +	1372.00 +	754.00 +	22.10 +
HBeAb, COI (>1.0)	6.79 −	3.93 −	2.74 −	0.82 +
HBcAb(T), COI (>1.0)	0.01 +	0.01 +	0.01 +	0.01 +

HBsAg, hepatitis B surface antigen; HBsAb, hepatitis B surface antibody; HBeAg, hepatitis B e antigen; HBeAb, hepatitis B e antibody; HBcAb(T), hepatitis B core total antibody; COI, cut-off index; + = positive; − = negative.

**Table 2:** Laboratory parameters in the case patient.

Test Date(yyyy-mm)	2023-08	2023-11	2023-12	2024-01	2024-03	2024-05	2024-06	2024-10	2025-04
HBV-DNA, IU/mL (<100)	/	8.52 × 10^2^	1.64 × 10^2^	3.35 × 10^2^	<100.00	9.25 × 10^2^	1.00 × 10^2^	<100.00	<100.00
ALT, U/L (<49)	51	46	48	53	51	44	75	65	23
AST, U/L (<40)	45	43	46	42	43	39	53	42	32
TB, μmol/L (5–23)	31.9	16.4	16.9	9.5	23.2	36.0	27.0	45.6	43.5
DBIL, μmol/L (<8.0)	9.6	5.5	5.9	3.2	8.1	11.8	10.1	14.5	13.6
IDIL, μmol/L (<17.0)	22.3	10.9	11.0	6.3	15.1	24.2	16.9	31.1	29.9
TP, g/L (62–76)	74.1	76.1	79.9	76.6	77.6	75.2	74.5	77.0	69.2
ALB, g/L (38–54)	46.1	48.0	51.6	47.5	48.4	47.0	46.4	46.6	46.4
GLB, g/L (20–40)	28.0	28.1	28.3	29.1	29.2	28.2	28.1	30.4	22.8
A/G (1.2–2.4)	1.6	1.7	1.8	1.6	1.7	1.7	1.7	1.5	2.0
GGT, U/L (<60)	12	9	8	8	11	9	10	11	8
ALP, U/L (91.1–278.1)	213	213	208	183	182	227	244	260	362
LDH, U/L (120–246)	269	337	303	272	270	279	288	273	254
PA, mg/L (200–430)	177	167	167	166	208	206	181	179	221
ADA, U/L (4–24)	16.7	17.6	17.0	18.9	15.8	16.6	16.5	18.3	14.8
BUN, mmol/L (3.2–8.2)	5.60	/	/	/	/	/	/	3.60	4.60
Cr, μmol/L (35.9–83.1)	60	/	/	/	/	/	/	52	49
UA, μmol/L (220–420)	358	/	/	/	/	/	/	277	278
CYSC, mg/L (<1.02)	0.82	/	/	/	/	/	/	0.51	0.88
P, mmol/L (1.45–2.1)	1.39	/	/	/	/	/	/	1.39	1.66
K+, mmol/L (3.5–5.5)	4.30	/	/	/	/	/	/	4.90	4.50
Na+, mmol/L (132–146)	137.8	/	/	/	/	/	/	138.8	138.6
Cl−, mmol/L (99–110)	103.3	/	/	/	/	/	/	102.7	105.3
Ca, mmol/L (2.25–2.67)	2.39	/	/	/	/	/	/	2.46	2.36
Mg, mmol/L (0.53–1.11)	0.83	/	/	/	/	/	/	0.87	0.86
SI, μmol/L (10.6–36.7)	23.80	/	/	/	/	/	/	4.30	21.40

HBV, hepatitis B virus, ALT, alanine transaminase; AST, aspartate transaminase; TB, total bilirubin; DBIL, direct bilirubin; IDIL, indirect bilirubin; TP, total protein; ALB, albumin; GLB, globulin; A/G, ratio of albumin to globulin; GGT, γ-glutamyl transpeptadase; ALP, alkaline phosphatase; LDH, lactate dehydrogenase; PA, prealbumin; ADA, adenosine deaminase; BUN, blood urea nitrogen; Cr, creatinine; UA, uric acid; CYSC, cystatin C; SI, serum iron.

## Data Availability

Data will be made available upon request.
